# Deficiency of the Immunoproteasome LMP10 Subunit Attenuates Angiotensin II-Induced Cardiac Hypertrophic Remodeling via Autophagic Degradation of gp130 and IGF1R

**DOI:** 10.3389/fphys.2020.00625

**Published:** 2020-06-09

**Authors:** Wen Yan, Zhi-Chao Dong, Jing-Jing Wang, Yun-Long Zhang, Hong-Xia Wang, Bo Zhang, Hui-Hua Li

**Affiliations:** ^1^Department of Emergency Medicine, Beijing Key Laboratory of Cardiopulmonary Cerebral Resuscitation, Beijing Chaoyang Hospital, Capital Medical University, Beijing, China; ^2^Department of Cardiology, Institute of Cardiovascular Diseases, First Affiliated Hospital of Dalian Medical University, Dalian, China; ^3^Department of Laboratory Animal Sciences, School of Basic Medical Sciences, Capital Medical University, Beijing, China; ^4^Department of Physiology and Physiopathology, School of Basic Medical Sciences, Capital Medical University, Beijing, China

**Keywords:** cardiac hypertrophy, immunoproteasome subunit, LMP10, ATG7, autophagy, gp130, IGF1R

## Abstract

**Background/Aim:**

Hypertensive cardiac hypertrophy is the leading cause of cardiac remodeling and heart failure. We recently demonstrated that the immunoproteasome, an inducible form of the constitutive proteasome, plays a critical role in regulating cardiovascular diseases. However, the role of the immunoproteasome LMP10 (β2i) catalytic subunit in the regulation of angiotensin II (Ang II)-induced cardiac hypertrophic remodeling remains unclear.

**Methods:**

Wild-type (WT) and LMP10 knockout (KO) mice were infused with Ang II 1,000 ng/kg/min for 2 weeks. Blood pressure was measured using a tail-cuff system. Cardiac function and hypertrophic remodeling were examined by echocardiography and histological staining. The expression levels of genes and proteins were examined with quantitative real-time PCR and immunoblotting analysis, respectively.

**Results:**

LMP10 mRNA and protein expression was significantly increased in Ang II-stimulated hearts and primary cardiomyocytes. Moreover, Ang II infusion for 2 weeks increased systolic blood pressure, abnormal cardiac function, hypertrophy, fibrosis, and inflammation in WT mice, which were significantly reversed in KO mice. Moreover, a marked reduction in the protein levels of insulin growth factor-1 receptor (IGF1R), glycoprotein 130 (gp130), and phosphorylated AKT, mTOR, STAT3, and ERK1/2 and an increase in the LC3II/I ratio were also observed in LMP10 KO mice compared with WT mice after Ang II infusion. *In vitro* culture experiments confirmed that LMP10 knockdown activated autophagy and increased IGF1R and gp130 degradation, leading to the inhibition of cardiomyocyte hypertrophy. However, inhibiting autophagy with chloroquine reversed this effect.

**Conclusion:**

The results of this study indicate that LMP10 KO attenuates Ang II-induced cardiac hypertrophic remodeling via the autophagy-dependent degradation of IGF1R and gp130, and suggests that LMP10 may be a novel therapeutic target for hypertrophic heart diseases.

## Introduction

Sustained cardiac hypertrophy is associated with a significant increase in the risk for heart failure and sudden death (Frey and Olson, 2003). Cardiomyocyte hypertrophy is the cellular response to a variety of extrinsic and intrinsic stimuli. The pathological changes of hypertrophy are characterized by myocyte growth, fibrosis, enhanced protein synthesis, and fetal gene expression (Frey and Olson, 2003). Neurohormonal stimuli such as angiotensin II (Ang II) play important roles in the pathogenesis of cardiac remodeling in a variety of diseases (Wang et al., 2016; Wang L. et al., 2018). Increasing evidence has demonstrated that Ang II can activate G protein-coupled receptors, receptor tyrosine/serine/threonine kinases, and cytokine/growth factor receptors, thereby stimulating intracellular signal transduction pathways that play an important role in the initiation, regulation, and adaptation of cardiac hypertrophy (Frey and Olson, 2003). Among these receptors, insulin growth factor-1 receptor (IGF1R) and glycoprotein 130 (gp130) modulate cell proliferation and differentiation through the constitutive activation of the PI3K/AKT, MAPK/ERK, and JAK/STAT3 pathways. Interestingly, IGF1R and gp130 are activated in hypertrophic hearts and contribute to the initiation of cardiac hypertrophy and heart failure as a response to pathological hypertrophic stress (Toyozaki et al., 1993; Hirota et al., 1995, 1999; Pan et al., 1998; Yasukawa et al., 2001; Matsui et al., 2002, 2003). Thus, strategies that modulate the expression and activation of IGF1R and gp130 may be promising approaches for the treatment of hypertrophic heart diseases.

The two major intracellular protein degradation pathways, the ubiquitin-proteasome system (UPS) and the autophagy-lysosomal system, play critical roles in the development of various diseases such as cancer and neurodegenerative and cardiovascular diseases (Powell and Divald, 2010; Xie, 2010; Li Z. et al., 2015). The UPS represents the major pathway for the selective degradation of short-lived and abnormal proteins (Powell and Divald, 2010; Xie, 2010). In contrast, autophagy primarily degrades long-lived proteins, such as receptors, and maintains amino acid pools in the setting of chronic starvation (Korolchuk et al., 2010). The 26S proteasome is the key component of the UPS and comprises two subcomplexes, the 20S proteasome and two 19S regulatory particles. The β1, β2, and β5 subunits of the proteasome perform caspase-like, trypsin-like, and chymotrypsin-like proteolysis, respectively (Angeles et al., 2012; Ferrington and Gregerson, 2012). Interestingly, under cytokine stimulation, such as interferon-γ, three additional catalytic β subunits, namely β1i (LMP2), β2i (LMP10 and MECL1), and β5i (LMP7) are induced and preferentially incorporated during proteasome assembly to form the immunoproteasome (Angeles et al., 2012; Ferrington and Gregerson, 2012). It is now clear that dysregulation of the immunoproteasome is associated with many human diseases, including cancer and autoimmune, neurodegenerative, cancer, and cardiovascular diseases (Angeles et al., 2012; Ferrington and Gregerson, 2012). Our recent data and other reports showed that the physiological low level and activity of immunoproteasome β subunits that are expressed in cardiac tissues are highly upregulated in response to hypertrophic stimuli such as Ang II, deoxycorticosterone acetate (DOCA)-salt, and pressure overload (Ferrington and Gregerson, 2012). In contrast, blockage of proteasome activity using an inhibitor represses cardiac hypertrophy (Li N. et al., 2015). Recently, we demonstrated that the upregulation of LMP7 contributes to the development of several cardiovascular diseases, including pressure overload-induced cardiac hypertrophy, Ang II-induced atrial fibrillation, abdominal aortic aneurysm, and retinopathy (Li F. D. et al., 2019; Li J. et al., 2019; Xie et al., 2019). Moreover, LMP10 plays a critical role in DOCA-salt-induced myocardial fibrosis (Yan et al., 2017); however, the role of LMP10 in the development of Ang II-induced cardiac hypertrophic remodeling remains unclear.

In this study, we discovered that LMP10 expression was significantly upregulated in Ang II-stimulated cardiomyocytes and hypertrophic hearts. Knockout (KO) of LMP10 markedly attenuated cardiac hypertrophic remodeling and improved adverse contractile function in mice. Mechanistically, LMP10 deficiency activated autophagy, which promoted the degradation of IGF1R and gp130, thereby inhibiting cardiac hypertrophy. Thus, our data suggest that LMP10 plays a critical role in modulating cardiac hypertrophic remodeling, and targeting LMP10 may be a new therapeutic approach for the treatment of hypertrophic diseases.

## Materials and Methods

### Animal Models

Wild-type (WT) C57BL/6 and LMP10 KO mice were initially obtained from Jackson Laboratory (Bar Harbor, ME, United States). Male mice (8–10 weeks old) were infused with saline or Ang II (Sigma-Aldrich, St. Louis, MO, United States) at a dose of 1,000 ng/kg/min using osmotic mini-pumps (Alzet, Cupertino, CA, United States) as described in our previous work (Wang L. et al., 2018). Blood pressure was measured in conscious mice by using a tail-cuff system (BP2010A; Softron, Tokyo, Japan) after Ang II infusion (Wang et al., 2016; Zhang et al., 2019). All animals were kept in a pathogen-free facility at Capital Medical University. All procedures were approved by the Institutional Animal Care and Use Committee of Capital Medical University and performed in accordance with the National Institutes of Health Guide for the Care and Use of Laboratory Animals.

### Cell Culture and Transfection

Neonatal rat cardiomyocytes (NRCMs) were obtained from 1–3-day-old Sprague-Dawley rats as described previously (Li et al., 2004; Chen et al., 2019). NRCMs were transfected with small interfering RNA (siRNA) against LMP10 (siRNA-LMP10) or scramble control (siRNA-control) for 24 h. To induce a hypertrophic response, NRCMs were treated with Ang II (100 nM) for 24 h, as described previously (Xie et al., 2019).

### Echocardiographic Assessment

All animals were lightly anesthetized with 1.5% isoflurane. Cardiac left ventricular (LV) structure and function were measured by M-mode echocardiography by using a 30-MHz probe (Vevo 2100 System; VisualSonics, Toronto, ON, Canada). LV inner diameter (LVID) and LV posterior wall thickness (LVPW) were measured at systole and diastole. LV ejection fraction (EF%) and LV fractional shortening (FS%) were calculated as follows: 100 × [(LVEDV − LVESV)/LVEDV] (%) and 100 × [(LVDd − LVDs)/LVDd] (%), respectively (Wang L. et al., 2018; Xie et al., 2019).

### Histopathological Examinations

Heart samples were fixed in a 4% formalin solution overnight, embedded in paraffin, and cut into 5-μm sections. Hematoxylin and eosin (H&E), wheat germ agglutinin (WGA) and Masson’s trichrome staining were performed as described previously (Wang L. et al., 2018; Xie et al., 2019). Images were taken at ×100 or ×200 magnification of 15–20 random fields from each heart sample. Myocyte size, fibrotic areas, and Mac-2-positive cells were analyzed by Image Pro Plus 3.0 (Nikon, Tokyo, Japan).

### Immunostaining

Neonatal rat cardiomyocytes were transfected with siRNA-control or siRNA-LMP10 for 24 h and stimulated with Ang II (100 nM) for an additional 24 h. Double immunostaining was performed with an anti-α-actinin, anti-LC3B, anti-IGF1R, or anti-gp130 antibody, and nuclei were stained with DAPI (blue). Cardiomyocyte surface area was measured in 150 cells in each experiment as described previously (Wang L. et al., 2018; Xie et al., 2019).

### Immunogold Electron Microscopy

Neonatal rat cardiomyocytes were fixed in 0.1% glutaraldehyde (Polysciences, Inc., Warrington, PA, United States) and 4% paraformaldehyde in 0.1 mol/L cacodylic acid for 30 min. The samples were probed with a mouse monoclonal antibody against IGF1R or gp130 (1:200 dilution) and viewed on an HT-7700 transmission electron microscope (Hitachi, Tokyo, Japan) as described previously (Swanlund et al., 2010).

### Immunoblotting Analysis

Heart tissues or NRCMs were lysed with RIPA lysis buffer (Solarbio, Beijing, China). Proteins (50–60 μg) were separated by sodium dodecyl sulfate-polyacrylamide gel electrophoresis, transferred to polyvinylidene difluoride membranes, and incubated with primary antibodies against AKT, phosphorylated (p)-AKT, mTOR, p-mTOR, STAT3, p-STAT3, ERK1/2, p-ERK1/2, calcineurin A, PTEN, and GAPDH (Cell Signaling Technologies, Boston, MA, United States), MKP-1 (Santa Cruz Biotechnology, Inc., Dallas, TX, United States), and LMP10 (Abcam, Cambridge, United Kingdom) as indicated in each experiment, and then with horseradish peroxidase-conjugated secondary antibodies (1:2,500) as described previously (Wang L. et al., 2018; Xie et al., 2019). Blot signal intensities were analyzed using a Gel-pro 4.5 Analyzer (Media Cybernetics, Rockville, MD, United States).

### Quantitative Real-Time PCR Analysis

Quantitative real-time PCR was conducted using an S1000 Thermal Cycler (Bio-Rad, Hercules, CA, United States). Total RNA was extracted from heart tissue with TRIzol (Invitrogen, Carlsbad, CA, United States) and reverse transcribed. cDNA (1–2 μg) was used for PCR amplification with gene-specific primers for LMP10, B-type natriuretic peptide (BNP), β-myosin heavy chain (β-MHC), interleukin (IL)-1β, IL-6, monocyte chemoattractant protein 1 (MCP-1), collagen I, and collagen III as described previously (Wang L. et al., 2018; Zhang et al., 2019). The amount of detected mRNA was normalized to the amount of endogenous GAPDH control.

### Proteasome Activity

Proteins were isolated from heart tissue with HEPES buffer (50 mM, pH 7.5) containing 20 mmol/L KCl, 5 mmol/L MgCl_2_, and 1 mmol/L DTT. Proteasome trypsin-like activity was measured in heart tissue using the fluorogenic peptide substrate Ac-RLRAMC (40 μmol/L) with excitation at 380 nm and emission at 460 nm as described previously (Chen et al., 2019).

### Statistical Analysis

Results are presented as the mean ± SEM. All statistical tests were performed using SPSS version 19.0. If each group satisfied normality and the variance among the groups was equal, differences in means for continuous variables were compared with Student’s *t*-test (two groups) or ANOVA (multiple groups). If these conditions were not met, a non-parametric Mann–Whitney U test was used. *P*-values < 0.05 were considered significant.

## Results

### Ang II Upregulates LMP10 Expression in Primary Cardiomyocytes and Mouse Hearts

**FIGURE 1:**
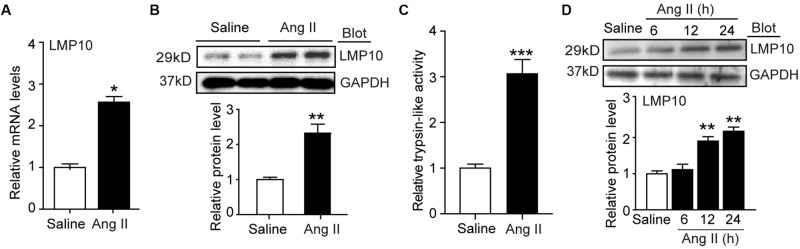
LMP10 was upregulated in Ang II-treated hearts and cardiomyocytes. **(A)** Wild-type (WT) mice were infused with angiotensin II (Ang II) at dose of 1,000 ng/kg/min for 2 weeks. qPCR analysis of LMP10 mRNA expression in Ang II-infused mouse hearts (*n* = 6). **(B)** Immunoblotting analyses of LMP10 protein levels in the hearts after Ang II infusion (upper). Quantification of the relative protein level (lower; *n* = 4). **(C)** Measurement of proteasome trypsin-like activity in Ang II-infused mouse hearts (*n* = 6). **(D)** Immunoblotting analyses of LMP10 protein levels in neonatal rat cardiomyocytes (NRCMs) exposed to Ang II (100 nM) at different time points (upper; h: hour). Quantification of the relative protein level (lower; *n* = 3 independent experiments). Data are presented as mean ± SEM, and *n* represents number of samples per group. *P < 0.05; **P < 0.01 versus saline; ***P < 0.001 versus saline.

To investigate the role of LMP10 in the development of cardiac hypertrophy, we examined LMP10 expression in Ang II-infused hearts. After 3 weeks, quantitative real-time PCR analysis revealed that LMP10 mRNA expression was significantly increased in Ang II-infused hearts compared with control saline-treated hearts (Figure 1A). Moreover, immunoblotting confirmed increased LMP10 protein expression in Ang II-infused hearts (Figure 1B). Accordingly, the proteasome trypsin-like activity generated by LMP10 was also increased in Ang II-infused heart tissue (Figure 1C). In addition, Ang II (100 nM) treatment upregulated the expression of LMP10 protein in NRCMs in a time-dependent manner (Figure 1C). Thus, these results suggest that the upregulation of LMP10 in cardiomyocytes may play a role in Ang II-infused cardiac hypertrophy.

### LMP10 Knockout Improves Ang II-Induced Contractile Function Abnormality and Cardiac Hypertrophy

**FIGURE 2:**
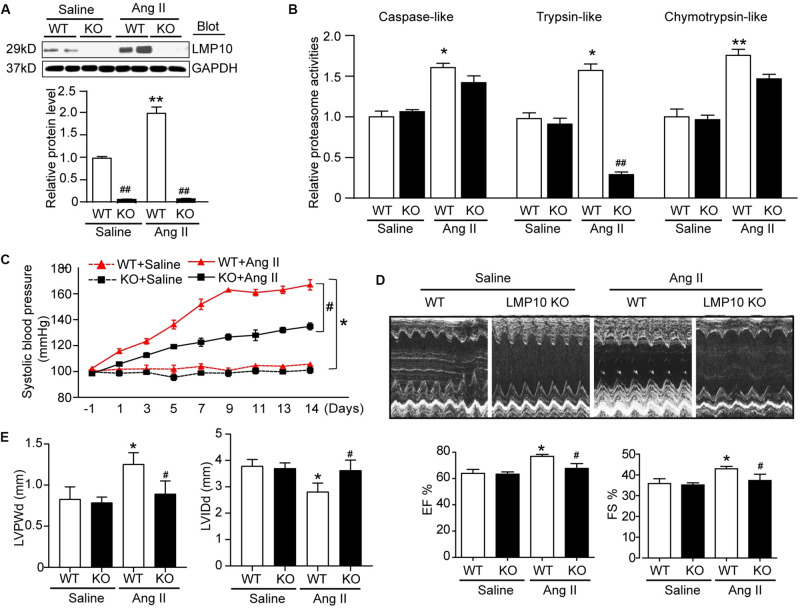
Knockdown of LMP10 ameliorates cardiac function in mice after Ang II infusion. Wild-type (WT) or LMP10 knockout (KO) mice were infused with angiotensin II (Ang II) at dose of 1,000 ng/kg/min for 2 weeks. **(A)** Immunoblotting analyses of LMP10 protein levels in the hearts (upper). Quantification of the relative protein level (lower; *n* = 6). **(B)** Measurement of proteasome caspase-like, trypsin-like, and chymotrypsin-like activities in the hearts (*n* = 6). **(C)** Representative M-mode echocardiography of left ventricular chamber. **(D)** Assessment of left ventricular ejection fraction (EF%) and fractional shortening (FS%) (*n* = 8). **(E)** Measurement of left ventricular inner diameter at end-diastole (LVIDd) and left ventricular posterior wall thickness at end-diastole (LVPWd) (*n* = 8). Data are presented as mean ± SEM, and n represents number of animals per group. *P < 0.05, **P < 0.01 versus saline; ^#^*P* < 0.05, ^##^*P* < 0.01 versus WT + Ang II.

**FIGURE 3:**
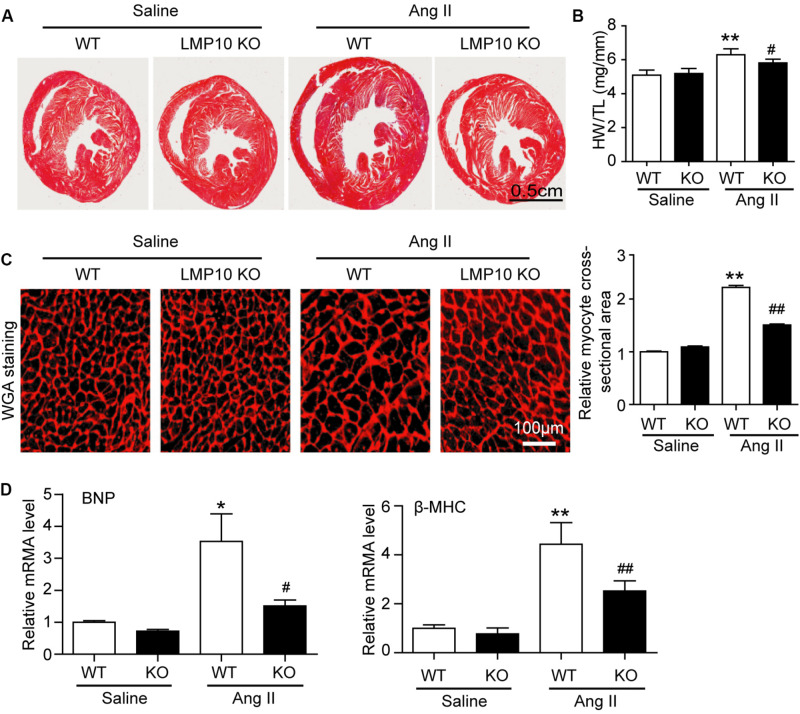
Deficiency of LMP10 attenuates Ang II-induced cardiac hypertrophy in mice. **(A)** Wild-type (WT) or LMP10 knockout (KO) mice were infused with angiotensin II (Ang II) at dose of 1,000 ng/kg/min for 2 weeks. Representative images of Hematoxylin and eosin (H&E) staining of the heart sections (lower). Scale bar 0.5 cm. **(B)** The ratios of heart weight to body weight (HW/BW) and heart weight to tibia length (HW/TL) (*n* = 6 per group). **(C)** TRITC-WGA staining of cardiac myocytes (left). Scale bar 100 μm. Quantification of the relative myocyte cross-sectional area (150–200 cells counted per heart, right) (*n* = 6 per group). **(D)** qPCR analyses of BNP and β-MHC mRNA levels in the hearts. Results are normalized to the GAPDH level (*n* = 6 per group). Data are presented as mean ± SEM, and n represents number of animals per group. *P < 0.05, **P < 0.01 versus saline; ^#^*P* < 0.05, ^##^*P* < 0.01 versus WT + Ang II.

To test the functional role of LMP10 in pathological hypertrophic remodeling, WT and LMP10 KO mice were infused with Ang II for 2 weeks. We found that Ang II infusion significantly increased LMP10 protein expression and systolic blood pressure in WT mice, whereas these increases were markedly attenuated in LMP10 KO mice (Figures 2A,B). Echocardiographic assessment reveled that the Ang II infusion-induced increase in cardiac contractile function, as reflected by an increased LV EF% and FS% in WT mice, was also significantly improved in LMP10 KO mice (Figure 2D). The Ang II-induced increase of LVPW was markedly reduced in LMP10 KO mice compared with WT control. The Ang II-induced decrease of left ventricular inner diameter at end-diastole (LVIDd) was also reversed in LMP10 KO mice (Figure 2E). Moreover, the features of Ang II-induced cardiac hypertrophy, as characterized by an increase in LV wall thickness (Figure 3A), heart weight/tibia length (HW/TL) ratios (Figure 3B), cross-sectional area of myocytes (Figure 3C), and atrial natriuretic peptide (ANP) and β-MHC mRNA expression (Figure 3D), were also remarkably attenuated in LMP10 KO mice (Figures 3A–D), suggesting that LMP10 exerts a prohypertrophic role *in vivo*.

### LMP10 Deficiency Inhibits Ang II-Induced Cardiac Fibrosis and Inflammation in Mice

**FIGURE 4:**
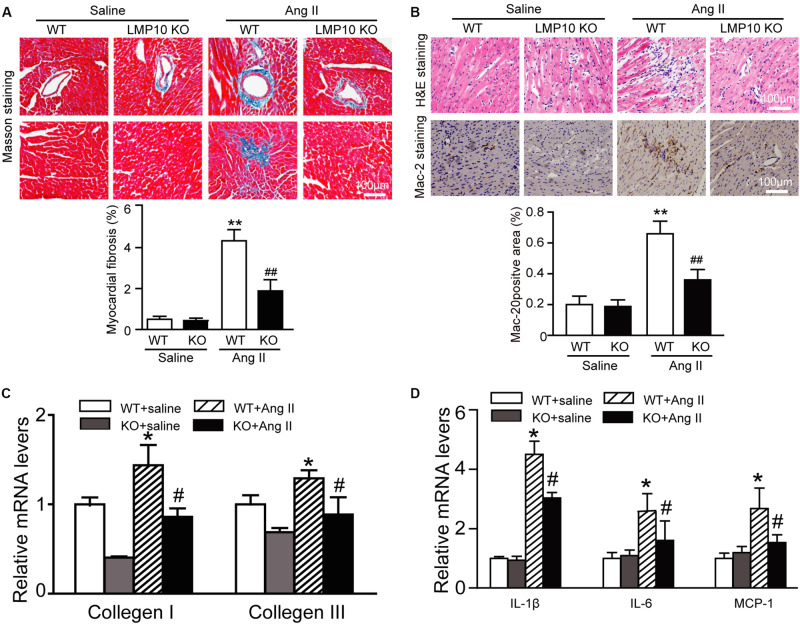
Deficiency of LMP10 attenuates Ang II-induced cardiac hypertrophy in mice. **(A)** Wild-type (WT) or LMP10 knockout (KO) mice were infused with angiotensin II (Ang II) at dose of 1,000 ng/kg/min for 2 weeks. Masson’s Trichrome staining of cardiac perivascular and interstitial fibrosis detected by (upper). Scale bar 100 μm. Quantification of the relative fibrosis area (lower, *n* = 6). **(B)** Hematoxylin and eosin (H&E) staining of the heart sections (upper). Immunochemical staining of heart sections with anti-Mac-2 antibody (middle). Scale bar 100 μm. Quantification of Mac-2-positive area (lower) (*n* = 6 per group). **(C)** qPCR analyses of collagen I and collagen III mRNA levels (*n* = 6). **(D)** qPCR analyses of IL-1β, IL-6 and MCP-1 mRNA levels (*n* = 6). The data are normalized to the GAPDH level. Data are presented as mean (SEM, and n represents number of animals per group. *P < 0.05, **P < 0.01 versus saline; ^#^*P* < 0.05, ^##^*P* < 0.01 versus WT + Ang II.

Myocardial fibrosis is a hallmark of cardiac remodeling; thus, we examined the extent of collagen deposition in the heart. Masson’s trichrome staining showed that Ang II infusion caused a significant increase in the myocardial perivascular and interstitial fibrotic areas and collagen I and III expression in WT mice, while this effect was markedly suppressed in Ang II-infused LMP10 KO mice (Figure 4A). Since inflammation is an important driver of myocardial fibrosis, we performed H&E and immunohistochemical staining. The Ang II infusion-induced accumulation of interstitial proinflammatory cells and Mac-2-positive macrophages in WT mice was evidently attenuated in Ang II-treated LMP10 KO mice (Figure 4B). Accordingly, the mRNA levels of collagen I, collagen III, IL-1β, IL-6, and MCP-1 were also obviously lower in LMP10 KO mice compared with WT mice after Ang II infusion (Figures 4C,D). No significant difference in these parameters was observed between the WT and LMP10 KO groups after saline infusion (Figures 4A–D).

### LMP10 Deficiency Reduces IGF1R and gp130 Protein Levels in Ang II-Infused Hearts

**FIGURE 5:**
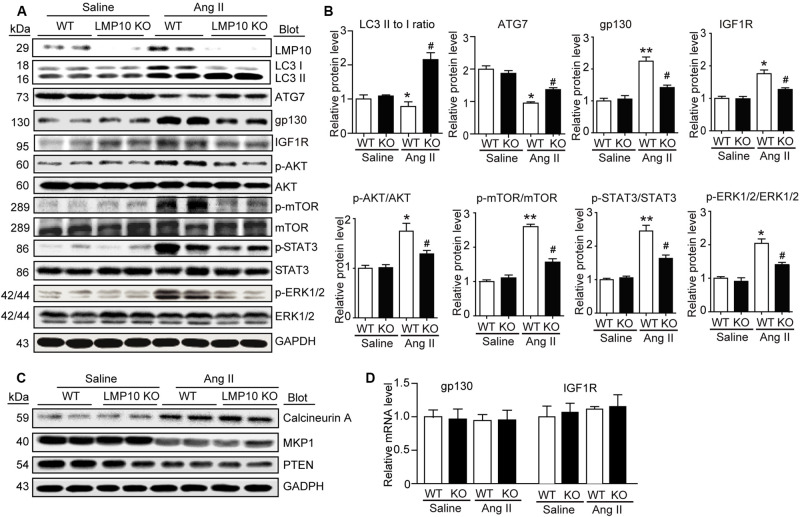
Deficiency of LMP10 reduces protein levels of IGF1R and gp130 and activation of the downstream mediators in Ang II-infused hearts. **(A)** Wild-type (WT) or LMP10 knockout (KO) mice were infused with angiotensin II (Ang II) at dose of 1,000 ng/kg/min for 2 weeks. Immunoblotting analyses of LMP10, LC3 II/I, ATG7, IGF1R, gp130, AKT, p-AKT, mTOR, p-mTOR, STAT3, p-STAT3, ERK1/2, and p-ERK1/2 protein levels in the heart tissues (left). **(B)** Quantification of the relative protein level (*n* = 4, right). **(C)** Immunoblotting analyses of calcineurin A, PTEN, and MKP-1 protein levels in the heart tissues. **(D)** qPCR analyses of IGF1R and gp130 mRNA levels (*n* = 6). The data are normalized to the GAPDH level. Data are expressed as the mean ± SEM. *P < 0.05, **P < 0.01 versus saline. ^#^*P* < 0.05 versus WT + Ang II.

To explore the molecular mechanism by which LMP10 KO attenuates Ang II-induced cardiac hypertrophy, we examined a range of prohypertrophic signaling pathways, including IGF1R, gp130, EGFR, and calcineurin A, and their downstream mediators. Immunoblotting revealed that LMP10 knockdown significantly increased the protein levels of LC3II/I ratio and ATG7 protein levels but reduced the protein levels of IGF1R, gp130, and phosphorylated AKT, mTOR JAK2, STAT3, and ERK1/2 compared with WT controls after Ang II treatment (Figures 5A,B). However, there was no significant difference in the protein levels of calcineurin A, MKP-1, and PTEN as well as the mRNA levels of IGF1R and gp130 between both groups after saline treatment (Figures 5C,D), suggesting that LMP10 is involved in the degradation of IGF1R and gp130 proteins.

### LMP10 Knockdown Activates Autophagy to Increase IGF1R and gp130 Degradation *in vitro*

**FIGURE 6:**
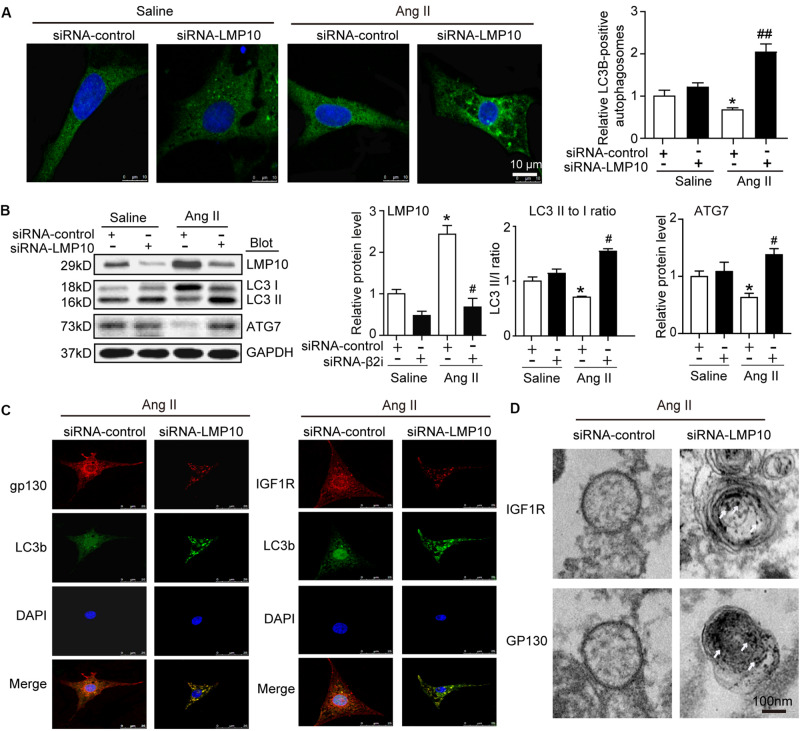
Knockdown of LMP10 increases autophagy and localization of IGF1R and gp130 in autophagosomes in cultured cardiomyocytes. Neonatal rat cardiomyocytes (NRCMs) were transfected with siRNA-LMP10 or siRNA-control for 24 h and then exposed to Ang II (100 nM) for 48 h. **(A)** Immunofluorescence staining of autophagosomes with anti-LC3B (green, left). DAPI staining for nuclei (blue). Quantification of LC3B-positive fluorescent dots (*n* = 10–12 cells per group, right). **(B)** Immunoblotting analysis of LC3 (upper) and ATG7 (middle). The ratios of LC3-II to LC3-I and quantitation of ATG7 protein level (*n* = 3 independent experiments). **(C)** Immunofluorescence staining for IGF1R or gp130 (red), and LC3B (green). DAPI staining for nuclei (blue) in NRCMs after Ang II. The data are normalized to the GAPDH level. **(D)** IGF1R and gp130 were respectively labeled using anti-IGF1R or gp130 antibody and the secondary antibody coupled to gold beads. Electron microscopic examination of EGFR and IGF1R in autophagosomes in NRCMs after Ang II. White arrows indicate IGF1R or GP130-positive particles. Data are expressed as the mean ± SEM. *P < 0.05 versus saline. ^#^*P* < 0.05, ^##^*P* < 0.01 versus WT + Ang II.

Increasing evidence suggests a link between the proteasome and autophagy in cancer and other cells (Korolchuk et al., 2010). Therefore, we tested whether LMP10 regulated the activation of autophagy in cardiomyocytes. Immunostaining showed that LMP10 knockdown by siRNA significantly increased the number of LC3-positive autophagosomes (a reliable marker of autophagosomes) in NRCMs after Ang II treatment compared with siRNA-control (Figure 6A). Furthermore, immunoblotting indicated that the conversion of LC3 I to LC3 II and protein level of ATG7 (markers for the activation of autophagy) were significantly higher in siRNA-LMP10-transfected NRCMs than in siRNA-control cells (Figure 6B).

To further evaluate whether LMP10 is required for the colocalization of IGF1R or gp130 with LC3B in cardiomyocytes, we performed immunofluorescence staining with an anti-IGF1R, anti-gp130, or anti-LC3B antibody. A smaller number of IGF1R- or gp130-positive vesicles colocalized within LC3B-positive autophagosomes in the siRNA-control group, but this colocalization was significantly increased in siRNA-LMP10-transfected NRCMs after Ang II treatment (Figure 6C). Electron microscopy further indicated that the number of immunogold-labeled IGF1R- or gp130-positive particles within autophagosomes was higher in siRNA-LMP10-transfected NRCMs than in siRNA-control cells after Ang II treatment (Figure 6D).

### LMP10 Knockdown Attenuates Cardiomyocyte Hypertrophy by Increasing IGF1R and gp130 Degradation

**FIGURE 7:**
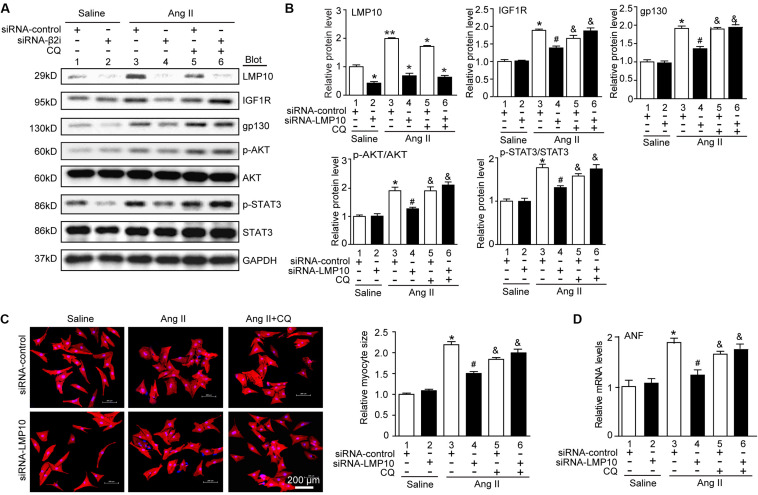
Knockdown of LMP10 inhibits cardiomyocyte hypertrophy through autophagic degradation of IGF1R and gp130 *in vitro*. **(A)** Immunoblotting analysis of protein levels of LMP10, IGF1R, gp130, p-AKT, AKT, p-STAT3, and STAT3 (*n* = 3). The data are normalized to the GAPDH level. **(B)** Quantification of the relative protein level (*n* = 4, right). **(C)** Neonatal rat cardiomyocytes (NRCMs) were transfected with siRNA-LMP10 or siRNA-control for 24 hours and then exposed to Ang II (100 nM) for 48 hours. Double immunostaining (red: α-actinin for cardiomyocytes; blue: DAPI for nuclei) of cardiomyocytes for measurement of cell size. Quantification of cardiomyocyte surface area (right, 150 cells counted per experiment, *n* = 3). Scale bar, 50 μm. **(D)** qPCR analysis of ANF mRNA expression (*n* = 3 independent experiments). Data are presented as means ± SEM. *P < 0.05 versus siRNA-control + saline; ^#^*P* < 0.05 versus siRNA-control + Ang II; ^&^*P* < 0.05 versus siRNA-control + Ang II.

To assess whether LMP10 regulates the degradation of IGF1R or gp130 via an autophagy-dependent pathway, NRCMs were transfected with siRNA-control or siRNA-LMP10 in the presence or absence of the autophagy inhibitor chloroquine (CQ). After Ang II treatment, LMP10 knockdown markedly reduced the protein levels of IGF1R, gp130, p-AKT, and p-STAT3 compared with Ang II alone (Figure 7A, lane 4 vs 3). This effect was fully reversed by CQ (Figure 7C, lane 5 vs lane 4), and by siRNA-LMP10 plus CQ (Figure 7C, lane 6 vs lane 5), indicating that that LMP10 regulates IGF1R and gp130 degradation via autophagy. Accordingly, LMP10 knockdown markedly attenuated Ang II-induced cardiomyocyte hypertrophy and ANP expression compared with Ang II stimulation (Figures 7D,E, lane 4 vs lane 3), whereas this effect was markedly restored by CQ and the combination of CQ with siRNA-LMP10 (Figures 7D,E, lane 5 vs lane 4 and lane 6 vs lane 5, respectively). Overall, these results demonstrate that LMP10 knockdown induces the autophagic degradation of IGF1R and gp130, which in turn inhibits cardiomyocyte hypertrophy.

## Discussion

**FIGURE 8:**
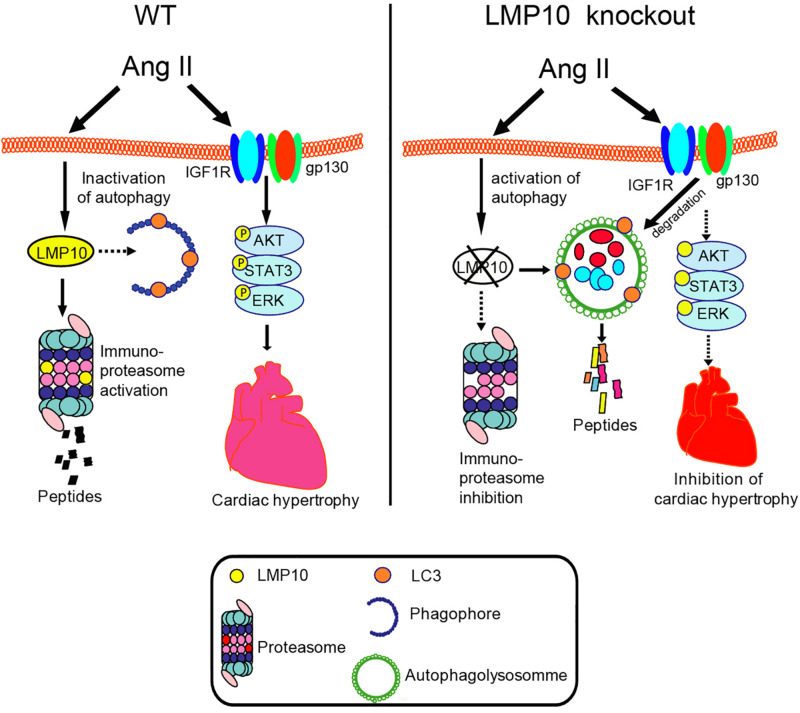
The summarized diagram showing that the proposed mechanisms underlying LMP10 regulate cardiac hypertrophy. Upon Ang II stimulation, upregulation of LMP10 inhibits autophagy activation, which then increases protein levels of IGF1R and gp130 and activation of the downstream mediators leading to cardiac hypertrophy. Conversely, LMP10 KO attenuates these effects.

Here, we provided new evidence that LMP10 exerts a prohypertrophic role *in vitro* and *in vivo*. We demonstrated that LMP10 expression was significantly upregulated in Ang II-infused hearts. LMP10 KO inhibited Ang II-induced cardiac hypertrophic remodeling and improved abnormal cardiac function. Mechanistically, the Ang II-induced upregulation of LMP10 inhibited the activation of autophagy, which in turn increased the stability of IGF1R and gp130 and subsequent development of cardiac hypertrophy. Therefore, our results revealed a functional link between LMP10 and autophagy in cardiomyocytes, and thus LMP10 may represent a therapeutic target for treating hypertrophic diseases. These data are summarized in Figure 8.

Previous studies have demonstrated that immunoproteasome dysregulation is associated with many human diseases, including cancer and autoimmune, and neurodegenerative diseases (Angeles et al., 2012; Ferrington and Gregerson, 2012). Recently, we reported that the immunoproteasome is involved in the regulation of several cardiovascular diseases. The functional roles of LMP7 in cardiac hypertrophy, atrial fibrillation, abdominal aortic aneurysm, and retinopathy have been investigated extensively (Li F. D. et al., 2019; Li J. et al., 2019; Xie et al., 2019; Wang et al., 2020). Meanwhile, we found that LMP10 (β2i) plays a critical role in DOCA-salt-induced myocardial fibrosis and high-dose Ang II-induced atrial fibrillation and retinopathy (Yan et al., 2017; Li J. et al., 2018; Wang S. et al., 2018). However, the elevation of blood pressure induced by a high dose of Ang II (2,000–3,000 ng/kg/min) in WT mice was not observed in LMP10 KO mice (Li J. et al., 2018; Wang S. et al., 2018). Interestingly, the present study showed that LMP10 KO markedly reduced medium-dose (1,000 ng/kg/min) Ang II-induced hypertension, but suppressed Ang II-induced cardiac hypertrophic remodeling and improved abnormal cardiac function (Figures 2–4), suggesting that LMP10 KO exerts a cardioprotective effect, likely through a blood-dependent mechanism. However, the mechanism by which LMP10 affects vascular remodeling and function needs to be determined in the future.

It is well documented that proteasomal inhibition usually increases autophagy, leading to the degradation of receptors in cancer and other cells (Korolchuk et al., 2010; Larrue et al., 2016). Although the exact mechanisms by which the proteasome regulates autophagy remain unknown, several potential mechanisms have been proposed. For example, the unfolded protein response induces ATF4 expression, which upregulates ATG5, ATG7, and LC3 expression or increases IRE1 and JNK1 levels, leading to Bcl-2 phosphorylation and the release of ATG6 (Ding et al., 2007; Milani et al., 2009; Zhu et al., 2010). Recently, we revealed that LMP7 can interact with and promote the degradation of ubiquitinated ATG5, which inhibits autophagy, thereby leading to pressure overload- or Ang II-induced cardiac hypertrophy (Xie et al., 2019). Here, we further showed that LMP10 KO in mice or LMP10 knockdown by siRNA in primary cardiomyocytes significantly increased the LC3II/I ratio, ATG7 protein level, and number of LC3B-positive particles (Figures 5A, 6A,B, respectively), indicating that LMP10 is a negative regulator of autophagy in cardiomyocytes.

Autophagy is a catabolic process essential for maintaining cardiac homeostasis in response to various forms of stresses. Autophagy is initiated by the formation of autophagosomes, which are regulated by ATG7 (E1 enzyme), ATG3 (E2 enzyme), ATG8 (also known as LC3, ubiquitin-like protein), ATG5, and ATG6 (Beclin 1) (Li Z. et al., 2015). Several studies have demonstrated that the dysregulation of autophagy is associated with cardiac hypertrophy (Nakai et al., 2007; Bhuiyan et al., 2013; Eisenberg et al., 2016). Increasing evidence suggests that autophagy exerts a cardioprotective effect through multiple mechanisms, including the degradation and recycling of long-lived proteins, lipid droplets, or damaged organelles, the clearance of reactive oxygen species, and the collaboration between autophagy and the UPS in protein quality control (Li Z. et al., 2015). It is well reported that IGF1R and gp130 mediate three major downstream pathways, JAK/STAT, Ras/MEK/ERK, and PI3K/AKT, which play different roles in myocardial infarction, cardiac hypertrophy, and heart failure (Toyozaki et al., 1993; Hirota et al., 1995; Pan et al., 1998; Hirota et al., 1999; Yasukawa et al., 2001; Matsui et al., 2002, 2003). Previous studies demonstrated that suppressor of cytokine signaling 3 (SOCS3) binds directly to gp130 and inhibits its downstream mediators (Yasukawa et al., 2001). A recent study suggested that the proteasome inhibitor YSY01A downregulates gp130 and the activation of JAK2 and STAT3 through a lysosome-autophagy pathway in cancer cells (Huang et al., 2017). Moreover, we recently demonstrated that the activation of autophagy by gallic acid induces the degradation of EGFR and gp130, leading to the inhibition of the downstream signaling cascades (Yan et al., 2019). In the present study, we further revealed that LMP10 KO induced autophagy, which promoted the colocalization of IGF1R and gp130 within autophagosomes and their subsequent degradation, leading to the inhibition of downstream mediators (mTOR, AKT STAT3, and ERK1/2) and cardiac hypertrophy (Figures 6, 7), indicating that LMP10 regulates cardiomyocyte hypertrophy via the autophagy-dependent degradation of IGF1R and gp130.

## Conclusion

Our data revealed a novel non-immune function for LMP10 in cardiac hypertrophy and dysfunction. We identified a regulatory mechanism by which LMP10 inhibited autophagy, leading to a reduction of IGF1R and gp130 degradation in cardiomyocytes. Our findings highlight the functional links between LMP10, autophagy, and receptors in the hypertrophic program of the heart. Further studies are needed to elucidate how LMP10 activates autophagy and how IGF1R and gp130 are degraded by autophagy in cardiomyocytes. Thus, our results suggest that targeting LMP10 may provide an approach for the treatment of hypertrophic diseases.

## Data Availability Statement

All datasets generated for this study are included in the article/supplementary material.

## Ethics Statement

All investigations were approved by and performed in accordance with the Animal Care and Use Committee of Capital Medical University, and conformed to the Guide for the Care and Use of Laboratory Animals published by the U.S. NIH.

## Author Contributions

WY, Z-CD, J-JW, Y-LZ, and H-XW conceived of the experiments, the acquisition of the data and analysis, and interpreted the data. WY and Z-CD participated in the statistical analysis of the primary data. H-HL and BZ drafted the manuscript and provided funding to support the study. H-HL supervised the study. All authors approved the final version of the manuscript.

## Conflict of Interest

The authors declare that the research was conducted in the absence of any commercial or financial relationships that could be construed as a potential conflict of interest.
